# Association of short-term nitrogen dioxide exposure with hospitalization for urolithiasis in Xinxiang, China: a time series study

**DOI:** 10.1007/s11356-023-28539-0

**Published:** 2023-07-29

**Authors:** Yangdong Li, Yongbin Wang, Maochuan Fan, Weisheng Li, Xiangzhen Meng, Hao Zhou, Shaohua Zhang, Qifeng Dou

**Affiliations:** 1 The First Affiliated Hospital of Xinxiang Medical University, No. 88, Jiankang Road, Weihui, Xinxiang, Henan Province 453100 People’s Republic of China; 2 Department of Epidemiology and Health Statistics, School of Public Health, Xinxiang Medical University, Xinxiang, Henan Province 453003 People’s Republic of China; 3 The First Affiliated Hospital of Henan University of Chinese Medicine, Zhengzhou, Henan Province 450003 People’s Republic of China

**Keywords:** Air pollution, Hospitalization, NO_2_, Time series analysis, Urolithiasis, Short-term exposure

## Abstract

Urolithiasis accounts for the highest incidence of all urologic-associated hospitalizations. However, few studies have explored the effect of nitrogen dioxide (NO_2_) on hospitalizations for urolithiasis. We included 5956 patients with urolithiasis, collected daily meteorological and air pollution data between 2016 and 2021, and analyzed the associations between air pollutants and hospitalization, length of the hospital stay, and hospitalization costs attributable to urolithiasis. NO_2_ exposure was associated with an increased risk of hospitalization for urinary tract stones. For each 10-μg/m^3^ increase and 1-day lag of NO_2_, the maximum daily effect on the risk of hospitalization for urolithiasis was 1.020 (95% confidence interval [CI]: 1.001–1.039), and the cumulative effect peaked on lag day 4 (relative risk [*RR*]: 1.061; 95% CI: 1.003–1.122). Attribution scores and quantitative analysis revealed that the mean number of hospital days and mean hospital costs were 16 days and 21,164.39 RMB, respectively. Up to 5.75% of all urolithiasis hospitalizations were estimated to be attributable to NO_2_, and the cost of NO_2_-related urolithiasis hospitalizations reached approximately 3,430,000 RMB. Stratified analysis showed that NO_2_ had a more sensitive impact on urolithiasis hospitalizations in women and in those aged ≥65 years. Notably, men and those younger than 65 years of age (exclude people aged 65) incurred more costs for urolithiasis hospitalizations. In the population level, the association between NO_2_ and risk of urolithiasis hospitalization was more pronounced during the warm season. NO_2_ can increase hospitalizations for urolithiasis for Xinxiang City residents, and there is a cumulative lag effect. Focusing on air pollution may have practical significance in terms of the prevention and control of urolithiasis.

**Supplementary Information:**

The online version contains supplementary material available at 10.1007/s11356-023-28539-0.

## Introduction

Urolithiasis is one of the most common and complex diseases of the urinary system worldwide, and its onset is related to age, sex, diet, and the environment. Currently several air pollutants, including particulate matter (PM_2.5_ and PM_10_), nitrogen dioxide (NO_2_), carbon monoxide (CO), ozone (O_3_), and sulfur dioxide (SO_2_), have been found to be associated with kidney disease. Nowadays, studies have confirmed a strong correlation between NO_2_ concentration and mortality, which is consistent with the long-term impact of PM_2.5_ on mortality (Atkinson et al. 2018). With the rapid development of the economy, issues associated with the atmospheric environment are increasingly drawing the public’s attention, particularly in recent years during which the frequent haze weather and environmental quality decline have caused a lot of adverse effects on people’s health. Moreover, industrial production processes, garbage incineration, and urban residential life are the main sources of NO_2_, and people’s lack of self-protection awareness from NO_2_ pollution will also have a certain impact on human health (Zhu et al. 2019).

In addition, most of the major air pollutants are strong oxidants that induce oxidative stress in human cells, which is a modifiable risk factor for cell damage. NO_2_ and O_3_ are the two most important gaseous air pollutants, and both have strong oxidation capacity. Therefore, exposure to NO_2_, O_3_, and PM is associated with the occurrence of oxidative stress in human body (Zhang et al. 2021; Afsar et al. 2019; Mohammad et al. 2021). At present, the concentration of NO_2_ in the atmosphere has become one of the indicators for measuring the strength of air pollution, which is associated with the occurrence and development of human diseases, such as chronic kidney (Wu et al. 2022), cardiovascular (Huang et al. 2021), and respiratory system (Liang et al. 2021) disease. However, little is known about the association between air pollutants and the risk of hospitalization for urinary stones. A better understanding of this association will be of great significance for the development of prevention and treatment strategies for urolithiasis.

Therefore, this study aimed to use the distributed hysteresis nonlinear model to analyze the nonlinear relationship and hysteresis effect between air pollutants and meteorological factors and the number of hospitalizations, number of hospitalization days, and hospitalization costs associated with urolithiasis in Xinxiang City, China, in order to provide a reference for reducing the influence of air pollutants on the risk of hospitalization of urolithiasis.

## Materials and methods

### Study area

This study was conducted in Xinxiang, China, which is located in the hinterland of the Central Plains in the northern part of Henan Province. The area has a warm temperate continental climate. Xinxiang is an important industrial base and central city in the Central Plains, and is a key city involved in the “2 + 26” air pollution monitoring program in the Beijing-Tianjin-Hebei region and surrounding areas.

### Study population and data source

We collected data of a total of 5956 hospitalizations for urolithiasis from the First Affiliated Hospital of Xinxiang Medical College, the Third Affiliated Hospital of Xinxiang Medical College, Xinxiang Maternal and Child Health Hospital, and the First People’s Hospital of Xinxiang City. The study period ranged from January 1, 2016 to October 31, 2021. The collected data included the number of hospitalizations attributable to urolithiasis, average number of days in the hospital, date of hospitalization, date of discharge, age, sex, and medical costs during hospitalization. The 10th edition of the *International Classification of Diseases* (ICD-10) was used to statistically organize urolithiasis (ICD-10 code range: N20–N23). Meteorological data, including the average daily temperature, average daily relative humidity, and average daily barometric pressure during the study period, were obtained from the National Meteorological Science Center (http://data.cma.cn/). The average daily mass concentrations of air pollutants, such as PM_2.5_, PM_10_, NO_2_, CO, SO_2_, and the daily maximum 8-h mean concentrations of ambient O_3_ during the study period, were obtained from the China Environmental Monitoring Station (http://www.cnemc.cn).

### Theory/calculation

The SPSS software (IBM, SPSS Inc., New York, NY, USA) was used for data processing and analyses. Descriptive statistics, such as the mean, minimum, maximum, 25th percentile (P_25_), 50th percentile (P_50_), and 75th percentile (P_75_), were used to describe the total number of hospitalizations for urolithiasis, the average length of the hospital stay (days), sex, age, meteorological factors, and air pollutants. The “DLNM,” “mgcv,” and “spline” packages of the R software (version 4.2.1) were used to construct the distributed lag nonlinear model (DLNM). The total number of hospitalizations for urolithiasis was considered as the dependent variable. A daily average temperature cross base was established, and the following were incorporated into the model: natural cubic spline function of long-term trends in pollutant data (PM_2.5_, PM_10_, NO_2_, SO_2_, CO, and O_3_), average daily relative humidity, time, day of the week effect, and holiday effect. This resulted in the following equation (Wu et al. 2022):

$$\textrm{Logit}\left[E\left({Y}_t\right)\right]=\alpha +\beta \times {X}_{\textrm{pollutant}}+ ns\left( time, df=7\times year\right)+ ns\left( temp, df=3\right)+ ns\left( humidity, df=3\right)+ dow+ holidays$$

In the above formula, *Y*_t_ is the number of hospitalizations for urolithiasis, *α* is the intercept, *β* is the regression coefficient, *X*_pollutant_ denotes the pollutant of interest, *ns* denotes the natural spline function, *df* is the degrees of freedom, *temp* is the temperature, *humidity* is the relative humidity, *dow* is the variable for the day of the week effect, and *holidays* is the holiday effect.

We estimated the economic loss associated with NO_2_ at the individual level for the attributable fraction (AF) and the attributable number (AN) of hospitalizations for urolithiasis, that is, the amount spent on urolithiasis per capita. Then, we evaluated the potential health and economic benefits of maintaining NO_2_ concentrations below the current NO_2_ air quality standard in China (24-h mean: 40 μg/m^3^) during the study period (Guo et al. 2019) using the following equation (Miettinen 1974):

$$PAF=P\left(E|D\right)\times \left( RR-1\right)/ RR$$

In the above equation, *P*(*E*|*D*) represents the proportion of exposure and disease in the population and *RR* represents the relative risk of exposure.

We stratified inpatients with urolithiasis according to season (warm season: May–October; cold season: November–April), age (younger than 64 years; 65 years or older), and sex. To assess the independence of the association of NO_2_ exposure with urolithiasis, we fitted a dual-pollutant model by introducing other gaseous air pollutants, including PM_2.5_, PM_10_, SO_2_, CO, and O_3_. In this study, a DLNM was used to analyze the nonlinear relationship between, and the lag effects on the risk of, hospitalization for urolithiasis and air pollutants and meteorological factors.

We calculated the effect estimate and 95% confidence interval (CI) for the association between NO_2_ exposure and urinary stones. *P* < 0.05 was considered statistically significant.

## Results

### Basic characteristics

A total of 5956 cases of urolithiasis were included in this study. Table 1 shows the descriptive statistics for urolithiasis, air pollutants, and meteorological factors during the study period. The median daily concentrations of PM_2.5_, PM_10_, NO_2_, CO, and SO_2_ were 46.00 μg/m^3^, 94.00 μg/m^3^, 40.00 μg/m^3^, 1.00 mg/m^3^, and 16.00 μg/m^3^, respectively, and the maximum 8-h concentration of O_3_ was 97.00 μg/m^3^. The average length of the hospital stay and average hospitalization cost across all urolithiasis cases were 16 days and 21,164.39 RMB, respectively. The median daily temperature was 17.10 °C, and the median daily relative humidity was 63.00%.

**Table 1:** Descriptive statistics of hospitalizations for urolithiasis, length of the hospital stay, hospitalization costs, air pollutants, and meteorological factors in Xinxiang, China, from January 1, 2016 to October 31, 2021

	$\overline{x}\pm s$	Minimum	25th	50th	75th	Maximum
Hospitalizations (counts)						
Total	2.79±2.18	0.00	1.00	2.00	4.00	15.00
Male	1.85±1.64	0.00	1.00	2.00	3.00	10.00
Female	0.95±1.10	0.00	0.00	1.00	1.00	8.00
<65 years	2.48±1.991	0.00	1.00	2.00	4.00	14.00
≥65 years	0.310±0.593	0.00	0.00	0.00	1.00	4.00
Hospitalizations (stays in days)						
Total	20.38 ±18.93	0.00	6.00	16.00	30.00	181.00
Male	12.83±14.19	0.00	1.00	9.00	19.00	139.00
Female	7.55± 10.73	0.00	0.00	3.00	11.00	96.00
<65 years	17.62± 16.97	0.00	5.001	14.00	25.00	181.00
≥65 years	2.76 ±6.43	0.00	0.00	0.00	1.00	84.00
Hospitalizations costs (RMB)						
Total	28,012.87 ±29,394.88	0.00	3746.05	21,164.39	41,533.24	310,457.87
Male	17,747.00± 21,831.64	0.00	558.80	10,859.96	27,771.75	273,340.76
Female	10,265.87 ±16,318.32	0.00	0.00	1644.50	16,204.45	109,953.05
<65 years	24,151.21 ±26,469.59	0.00	2440.00	18,104.86	35,537.17	310,457.87
≥65 years	3861.66±9767.81	0.00	0.00	0.00	0.00	105,821.50
Meteorological factors						
Mean temperature (°C)	16.23± 10.11	−8.00	7.36	17.10	25.50	34.60
Relative humidity (%)	61.26 ±18.11	13.00	48.00	63.00	75.26	100.00
Air pollutants						
PM_2.5_ (μg/m^3^)	61.04±47.27	5.00	32.00	46.00	75.00	644.00
PM_10_ (μg/m^3^)	111.70±71.46	12.00	66.00	94.00	136.50	823.00
NO_2_ (μg/m^3^)	42.32±19.94	8.00	27.00	40.00	55.00	168.00
CO (mg/m^3^)	1.54± 0.70	0.30	0.70	1.00	1.37	8.00
SO_2_ (μg/m^3^)	21.31± 16.88	3.00	11.00	16.00	25.00	156.00
O_3_ (μg/m^3^)	104.20 ±55.01	7.00	60.00	97.00	145.00	276.00

A Spearman correlation analysis was performed to analyze the correlation between various variables of air pollutants and meteorological factors. These correlations are provided in Table 2.

**Table 2:** Spearman correlation between daily meteorological factors and air pollutants

Pollutants	PM_2.5_	PM_10_	CO	NO_2_	SO_2_	O_3_	Temp	RH
PM_2.5_	1.000							
PM_10_	0.869**	1.000						
CO	0.750**	0.612**	1.000					
NO_2_	0.683**	0.723**	0.620**	1.000				
SO_2_	0.476**	0.583**	0.497**	0.622**	1.000			
O_3_	−0.400**	−0.329**	−0.415**	−0.409**	−0.114**	1.000		
Temp	−0.532**	−0.493**	−0.456**	−0.488**	−0.264**	0.800**	1.000	
RH	0.126**	−0.172**	0.178**	−0.154**	−0.457**	−0.145**	0.119**	1.000

*Temp*, temperature; *RH*, relative humidity

**P-value < 0.001

**Fig. 1:**
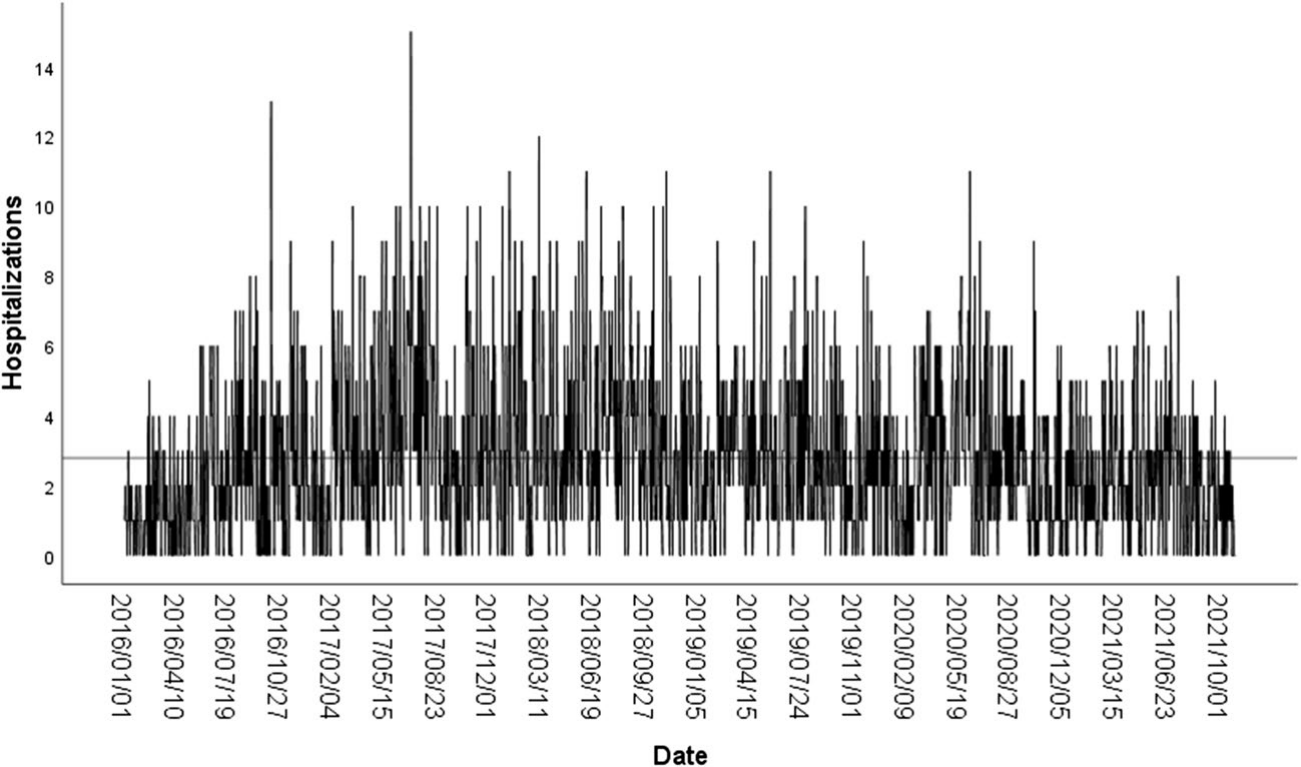
Time series of hospitalizations for urolithiasis in Xinxiang, China, from January 1, 2016 to October 31, 2021

In this study, from 2016 to October 31, 2021, there were 5956 hospitalizations for urolithiasis in Xinxiang, China. Of these hospitalizations, 3937 (66.10%) related to male and 2019 (33.90%) to female patients; therefore, the male-to-female ratio was approximately 1.95 (see Table 1). A time series plot of the number of hospitalizations for urolithiasis, length of the hospital stay, and hospitalization costs (Fig. 1; Figure S1) showed that the median number of hospitalizations per day was two. The total number of hospitalizations for urolithiasis was 5287 (88.77%) for those 65 years of age or younger. However, the total number of hospitalizations for urolithiasis was 669 (11.23%) for those older than 65 years of age.

### Effects of six air pollutants on hospitalizations for urinary tract stones

**Fig. 2:**
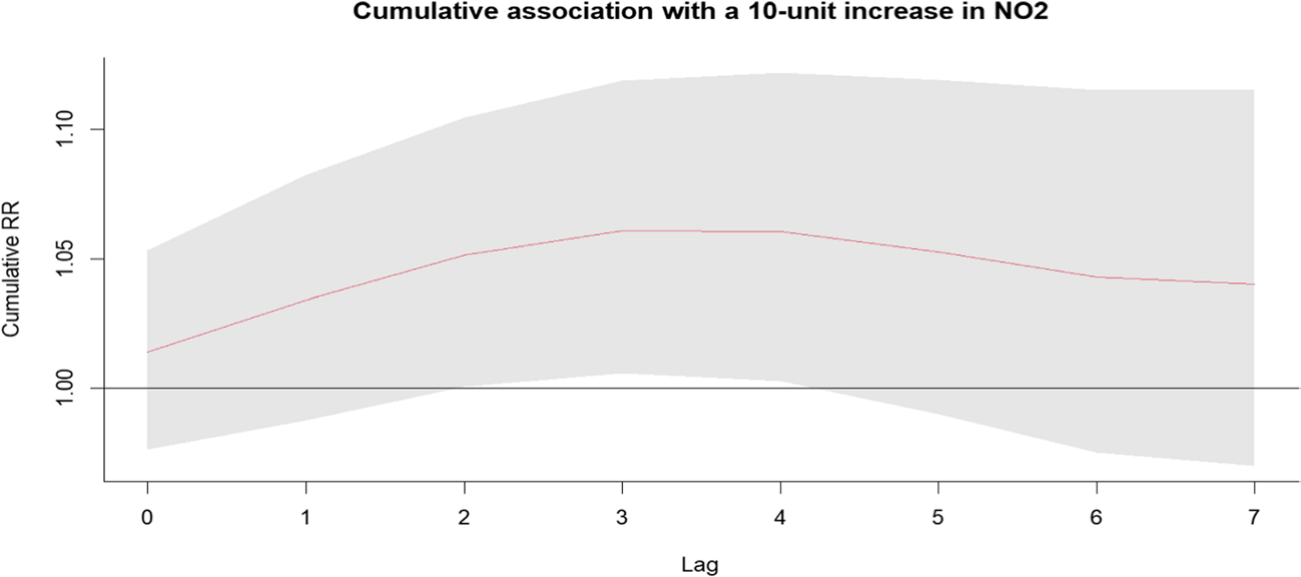
Cumulative effect of NO_2_ exposure on the number of hospitalizations for urolithiasis on different lag days

Using the single-pollutant model (see Table 3), we observed that the number of hospitalizations for urolithiasis increased with higher NO_2_ concentrations. Furthermore, it was observed that NO_2_ had a nonlinear relationship with the number of hospitalizations for urolithiasis (Fig. 2; Figure S2). The maximum single-day effect of NO_2_ on hospitalizations for urolithiasis was 1.020 (95% CI: 1.001–1.039 with a 1-day lag) for each 10-μg/m^3^ increase in the NO_2_ concentration. The cumulative effect on hospitalizations for urolithiasis peaked on lag day 4, with a relative risk (*RR*) of 1.061 (95% CI: 1.003–1.122). The results for the other pollutants are summarized in Fig. 2 and Figure S2.

**Table 3:** Single-day and cumulative lag effects of 10-μg/m^3^ increases in the concentrations of six pollutants on the number of hospitalizations for urolithiasis in Xinxiang, China, from January 1, 2016 to October 31, 2021

	PM_2.5_		PM_10_		NO_2_		SO_2_		CO		O_3_	
	RR	95% CI	RR	95% CI	RR	95% CI	RR	95% CI	RR	95% CI	RR	95% CI
Lag 0	1.012	(0.980–1.053)	0.999	(0.988–1.011)	1.014	(0.976–1.053)	0.993	(0.948–1.040)	1.025	(0.958–1.097)	0.991	(0.976–1.007)
Lag 1	1.016	(1.001–1.039)	1.000	(0.995–1.006)	1.020	(1.001–1.039)	1.006	(0.984–1.029)	1.002	(0.972–1.034)	1.005	(0.997–1.013)
Lag 2	1.014	(0.996–1.039)	1.000	(0.994–1.007)	1.017	(0.995–1.039)	1.010	(0.985–1.035)	0.993	(0.959–1.029)	1.009	(1.001–1.017)
Lag 3	1.008	(0.994–1.026)	1.000	(0.995–1.005)	1.009	(0.992–1.026)	1.008	(0.989–1.028)	0.994	(0.967–1.022)	1.008	(1.001–1.014)
Lag 4	1.000	(0.986–1.017)	1.000	(0.995–1.005)	1.000	(0.983–1.017)	1.003	(0.984–1.023)	1.000	(0.973–1.028)	1.003	(0.997–1.009)
Lag 5	0.994	(0.976–1.014)	1.001	(0.994–1.007)	0.993	(0.972–1.014)	0.999	(0.975–1.024)	1.007	(0.972–1.044)	0.999	(0.991–1.006)
Lag 6	0.992	(0.977–1.009)	1.002	(0.996–1.008)	0.991	(0.973–1.009)	0.998	(0.977–1.020)	1.012	(0.982–1.042)	0.998	(0.991–1.005)
Lag 7	0.998	(0.968–1.034)	1.005	(0.994–1.016)	0.997	(0.962–1.034)	1.004	(0.964–1.046)	1.009	(0.950–1.071)	1.004	(0.991–1.017)
Lag 0–1	1.028	(0.990–1.068)	0.999	(0.986–1.014)	1.034	(0.988–1.082)	0.999	(0.944–1.057)	1.027	(0.948–1.114)	0.996	(0.976–1.016)
Lag 0–2	1.043	(1.001–1.086)	1.000	(0.985–1.015)	1.051	(1.001–1.105)	1.009	(0.949–1.073)	1.021	(0.937–1.111)	1.005	(0.983–1.028)
Lag 0–3	1.050	(1.005–1.098)	1.000	(0.984–1.017)	1.061	(1.006–1.119)	1.017	(0.952–1.087)	1.015	(0.926–1.111)	1.013	(0.989–1.037)
Lag 0–4	1.050	(1.002–1.100)	1.000	(0.983–1.018)	1.061	(1.003–1.122)	1.021	(0.952–1.096)	1.015	(0.921–1.118)	1.016	(0.992–1.041)
Lag 0–5	1.044	(0.992–1.098)	1.001	(0.981–1.021)	1.053	(0.990–1.119)	1.020	(0.944–1.102)	1.022	(0.917–1.139)	1.015	(0.989–1.041)
Lag 0–6	1.036	(0.979–1.095)	1.003	(0.981–1.026)	1.043	(0.975–1.115)	1.018	(0.935–1.109)	1.034	(0.916–1.167)	1.013	(0.986–1.040)
Lag 0–7	1.033	(0.975–1.095)	1.008	(0.984–1.032)	1.040	(0.970–1.115)	1.022	(0.935–1.118)	1.043	(0.919–1.183)	1.016	(0.990–1.044)

Table 4 summarizes the results of the two-pollutant model for air pollutants. After adjusting for PM_2.5_, PM_10_, SO_2_, CO, and O_3_, the association between NO_2_ exposure (lag day 1) and hospitalizations for urolithiasis remained robust. Moreover, the correlation between NO_2_ (cumulative lag days 2, 3, and 4) and hospitalizations for urolithiasis was significantly enhanced after controlling for PM_2.5_, PM_10_, SO_2_, CO, and O_3_.

**Table 4:** Single-day and cumulative lag effects of 10-μg/m^3^ increases in NO_2_ and other air pollutant concentrations (PM_2.5_, PM_10_, SO_2_, CO, and O_3_) on hospitalizations for urolithiasis in a two-pollutant model from January 1, 2016 to October 31, 2021

	NO_2_ + PM_2.5_		NO_2_ + PM_10_		NO_2_ + SO_2_		NO_2_ + CO		NO_2_ + O_3_	
	RR	95% CI	RR	95% CI	RR	95% CI	RR	95% CI	RR	95% CI
Lag 0	1.021	(0.974–1.071)	1.022	(0.977–1.069)	1.028	(0.980–1.078)	1.011	(0.963–1.063)	1.019	(0.981–1.059)
Lag 1	1.041	(1.016–1.067)	1.033	(1.009–1.057)	1.027	(1.003–1.051)	1.035	(1.010–1.061)	1.020	(1.002–1.040)
Lag 2	1.040	(1.012–1.068)	1.029	(1.002–1.057)	1.019	(0.992–1.046)	1.036	(1.008–1.066)	1.015	(0.994–1.037)
Lag 3	1.024	(1.002–1.047)	1.016	(0.995–1.038)	1.007	(0.986–1.029)	1.022	(1.000–1.045)	1.007	(0.990–1.024)
Lag 4	1.002	(0.980–1.024)	1.000	(0.979–1.021)	0.996	(0.975–1.018)	1.002	(0.980–1.025)	0.999	(0.982–1.016)
Lag 5	0.981	(0.954–1.007)	0.985	(0.960–1.011)	0.987	(0.961–1.014)	0.985	(0.957–1.013)	0.992	(0.971–1.014)
Lag 6	0.967	(0.944–0.989)	0.977	(0.955–0.999)	0.985	(0.962–1.007)	0.977	(0.954–1.001)	0.991	(0.973–1.009)
Lag 7	0.966	(0.923–1.011)	0.979	(0.938–1.023)	0.991	(0.947–1.037)	0.988	(0.943–1.035)	0.997	(0.961–1.034)
Lag 0–1	1.063	(1.002–1.128)	1.056	(0.999–1.115)	1.056	(0.997–1.118)	1.047	(0.986–1.112)	1.040	(0.994–1.089)
Lag 0–2	1.105	(1.036–1.179)	1.086	(1.022–1.154)	1.076	(1.012–1.143)	1.085	(1.018–1.157)	1.056	(1.005–1.110)
Lag 0–3	1.132	(1.055–1.215)	1.104	(1.033–1.180)	1.084	(1.015–1.157)	1.109	(1.035–1.188)	1.064	(1.009–1.123)
Lag 0–4	1.134	(1.053–1.221)	1.103	(1.029–1.184)	1.079	(1.008–1.155)	1.111	(1.035–1.193)	1.063	(1.004–1.124)
Lag 0–5	1.112	(1.027–1.204)	1.087	(1.008–1.172)	1.065	(0.990–1.147)	1.094	(1.014–1.181)	1.054	(0.991–1.121)
Lag 0–6	1.075	(0.987–1.170)	1.062	(0.978–1.152)	1.049	(0.969–1.136)	1.070	(0.986–1.160)	1.044	(0.976–1.117)
Lag 0–7	1.039	(0.952–1.133)	1.040	(0.955–1.133)	1.039	(0.958–1.128)	1.057	(0.975–1.146)	1.041	(0.970–1.117)

### Attributable fraction and number

Other measures such as disease burden (attributable score and number), health burden (length of stay), and economic burden (cost of stay) were used to further understand the effect of NO_2_ on urolithiasis. In this study, we estimated that up to 5.75% of the total hospitalizations for urolithiasis could be attributed to NO_2_, and the cost of hospitalizations for urolithiasis related to NO_2_ amounted to approximately RMB 343 million (Table 5). It is worth noting that the magnitude of hospitalization risk for NO_2_ varies by sex, age, and season, indicating that the resulting burden of disease is significantly comparable. A bar chart was also used to analyze the distribution of continuous variables for hospitalizations, hospital stays, and hospitalization costs (supplementary Figure S3). Other measures such as disease burden (attributable score and number), health burden (length of stay), and economic burden (cost of stay) were used to further understand the effect of NO_2_ on urolithiasis disease.

**Table 5:** Fraction and number of urolithiasis hospitalizations, hospital stays, and hospitalization costs attributable to NO_2_ from January 1, 2016 to October 31, 2021

	Attributable fraction of hospitalizations (%)	Attributable number of hospitalizations (no.)	Attributable number of hospital stays (days)	Attributable number of hospitalization costs (RMB, million)
Total	5.75 (0.60–10.63)	342.47 (35.74–633.12)	2497.40 (260.60–4616.93)	3.43 (0.36–6.35)
Males	4.40 (−2.35–10.63)	173.23 (−92.52–418.50)	1203.14 (−642.58–2906.67)	1.66 (−0.89–4.02)
Females	3.19 (0.10–6.10)	64.41 (2.02–123.16)	513.24 (16.09–981.43)	0.70 (0.02–1.33)
Young (<65)	4.58 (−1.01–9.83)	242.14 (−53.40–519.71)	1719.38 (−379.16–3690.28)	2.36 (−0.52–5.06)
Elderly (≥65)	18.96 (5.12–30.80)	126.84 (34.25–206.05)	1117.12 (301.67–1814.74)	1.56 (0.42–2.53)
Warm	16.81 (3.75–28.11)	487.99 (108.86–816.03)	3550.27 (792.00–5936.83)	4.96 (1.11–8.30)
Cold	1.19 (−6.72–8.51)	36.33 (−205.16–259.81)	265.52 (−1499.43–1898.84)	0.36 (−2.03–2.57)

Note that figures in the parenthesis were the 95% confidence interval

### Effect of increased NO_2_ exposure on the number of hospitalizations for urolithiasis after stratification based on age, sex, and season

**Fig. 3:**
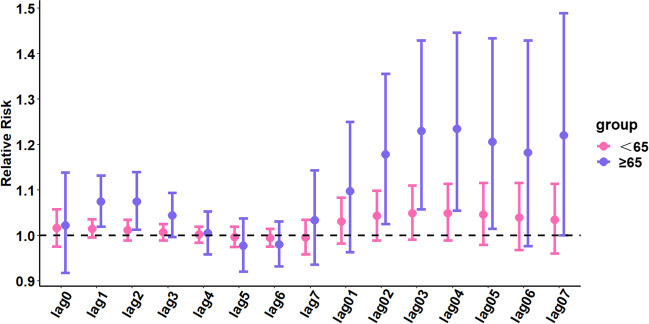
Hysteresis and cumulative hysteresis effects of each 10-μg/m^3^ increase in the NO_2_ concentration on the number of hospitalizations for urolithiasis on different lag days in the age-stratified analysis

The results of the age-stratified analysis (see Fig. 3) showed that for every 10-μg/m^3^ increase in the NO_2_ concentration, the correlation between age 65 years or older and the number of hospitalizations for urolithiasis was high. Additionally, the maximum single-day effect was 1.074 (95% CI: 1.012–1.139) on lag day 2, and the cumulative effect peaked at 1.234 (95% CI: 1.054–1.445) on lag day 4 (detailed results of the hierarchical analysis are shown in Table S1).

**Fig. 4:**
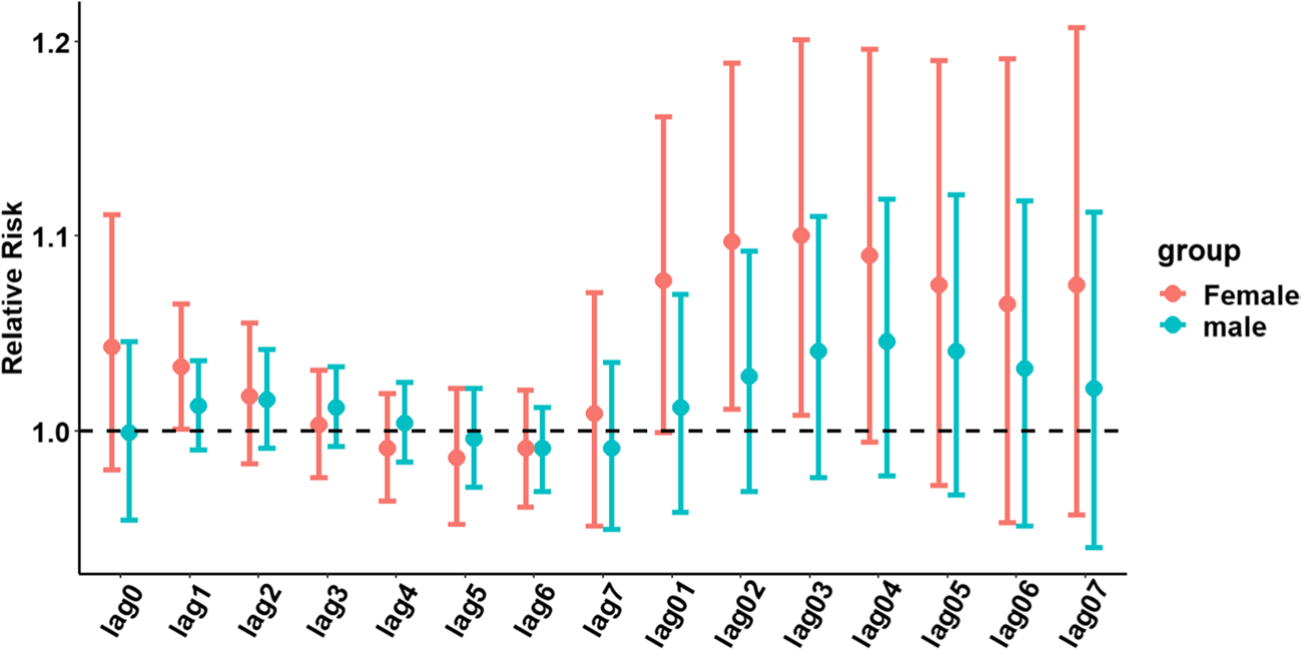
Hysteresis and cumulative hysteresis effects of each 10-μg/m^3^ increase in the NO_2_ concentration on the number of hospitalizations for urolithiasis on different lag days in the sex-stratified analysis

The results of the sex-stratified analysis (see Fig. 4) showed that for every 10-μg/m^3^ increase in the NO_2_ concentration, the correlation between female patients and hospitalizations for urolithiasis was high. Additionally, the maximum single-day effect was 1.033 (95% CI: 1.001–1.065) on lag day 1, and the cumulative effect peaked at 1.100 (95% CI: 1.008–1.201) on lag day 3 (detailed results of the hierarchical analysis are provided in Table S2).

**Fig. 5:**
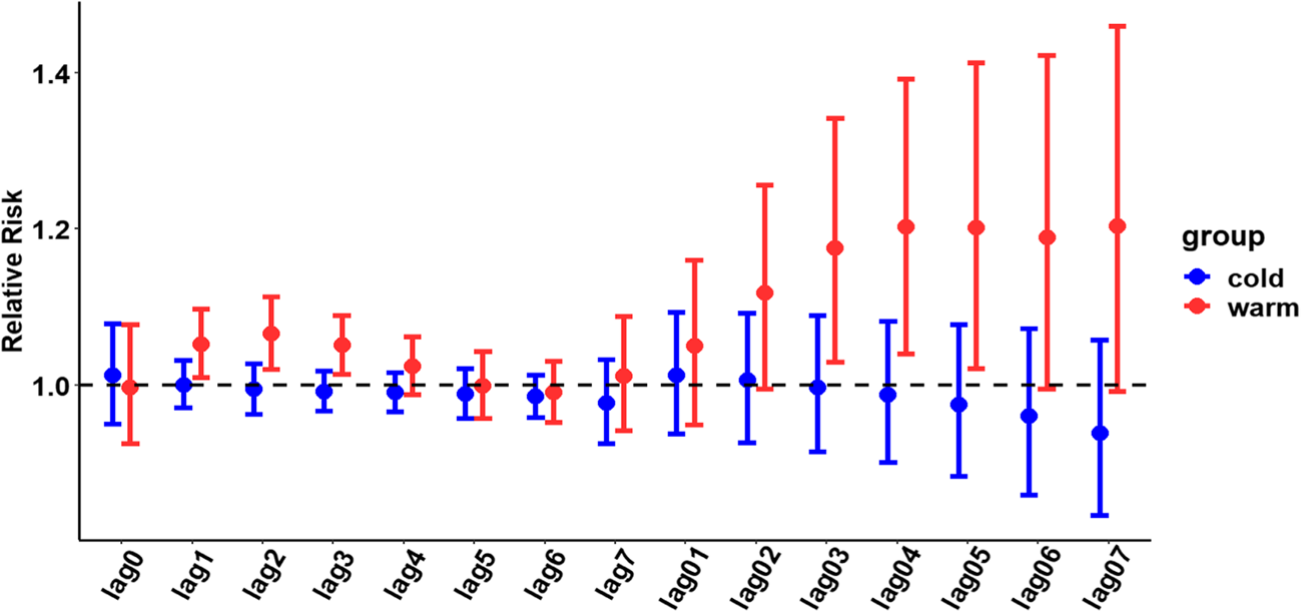
Lag and cumulative lag effects of each 10-μg/m^3^ increase in the NO_2_ concentration on the number of hospitalizations for urolithiasis on different lag days in the seasonal stratification analysis

The results of the seasonal stratification analysis (see Fig. 5) showed that the correlation between the warm season and the number of hospitalizations for urolithiasis was greater for each 10-μg/m^3^ increase in the NO_2_ concentration when the season was divided into cold and warm seasons. Additionally, the maximum single-day effect was 1.065 (95% CI: 1.020–1.113) on lag day 2, and the cumulative effect peaked at 1.202 (95% CI: 1.039–1.391) on lag day 4 (detailed results of the stratified analysis are provided in Table S3).

### Model stability analysis

By adjusting the degree of freedom of the natural spline function of time (8–13 df), it can be found that the relative risk of the model has no obvious change, so it can be considered that the established model has stability, and the results of model analysis are reliable (Table 6).

**Table 6:** Sensitivity analysis result

	Temperature degree of freedom	Male		Female		Young (<65)		Elderly (≥65)		Warm		Cold	
		RR	95% CI	RR	95% CI	RR	95% CI	RR	95% CI	RR	95% CI	RR	95% CI
df = 8	3	1.013	(0.990–1.037)	1.033	(1.001–1.066)	1.013	(0.993–1.034)	1.075	(1.019–1.134)	1.050	(1.006–1.095)	1.004	(0.972–1.037)
df = 9	3	1.011	(0.988–1.035)	1.028	(0.996–1.061)	1.011	(0.990–1.032)	1.072	(1.016–1.132)	1.046	(1.002–1.092)	0.999	(0.968–1.031)
df = 10	3	1.014	(0.990–1.038)	1.028	(0.995–1.062)	1.011	(0.990–1.032)	1.084	(1.025–1.146)	1.049	(1.003–1.096)	0.993	(0.961–1.026)
df = 11	3	1.008	(0.985–1.032)	1.024	(0.991–1.057)	1.006	(0.986–1.027)	1.074	(1.017–1.137)	1.050	(1.004–1.097)	0.997	(0.962–1.035)
df = 12	3	1.009	(0.986–1.034)	1.025	(0.993–1.059)	1.008	(0.987–1.029)	1.077	(1.019–1.139)	1.047	(1.000–1.095)	0.993	(0.954–1.032)
df = 13	3	1.009	(0.986–1.034)	1.023	(0.990–1.057)	1.008	(0.987–1.029)	1.072	(1.013–1.134)	1.056	(1.006–1.107)	1.008	(0.966–1.052)

## Discussion

This study used the DLNM model to investigate the association between air pollutants and daily mean temperature on the number of hospitalizations for urolithiasis, hospital length of stay, and hospitalization costs in Xinxiang City, China, from January 1, 2016 to October 31, 2021. The results showed a significant correlation between short-term NO_2_ exposure and the number of hospitalizations for urolithiasis. The correlation was more significant for elderly patients, female patients, and warm seasons. The association between NO_2_ exposure and urolithiasis was robust even after adjusting for other air pollutants.

Based on the DLNM analysis results, our study demonstrated that the number of daily hospitalizations in the time series presented a periodic distribution with the cold and warm seasons, which was higher in the warm (May to September) and lower in the cold (October to April of the next year) season. The concentration of various air pollutants also presented a cyclical trend, with O_3_, PM_2.5_, PM_10_, NO_2_, SO_2_, and CO being cyclically opposite with temperature, mainly high in the warm season and low in the cold season. In the single-pollutant model analysis, there was a nonlinear relationship between NO_2_ and the number of inpatients with urolithiasis, and there was a certain lag effect. This study found that NO_2_ exposure was associated with an increased risk of hospitalization for urolithiasis. At daily mean temperature, the maximum daily effect of NO_2_ and PM_2.5_ increased by 10 μg/m^3^ on the hospitalization risk of urolithiasis was 1 day behind, and the cumulative lag effect reached a peak on day 4. When O_3_ increased by 10 μg/m^3^, the maximum 1-day effect of O_3_ on the number of hospitalizations for urolithiasis was 2 days behind. However, no cumulative hysteretic effect of O_3_ on the number of hospitalizations for urolithiasis was found. No other association was observed between the concentrations of other pollutants (PM_10_, SO_2_, and CO) and the number of hospitalizations for urolithiasis per increase of 10 μg/m^3^ or 10 mg/m^3^. These results suggest that short-term NO_2_ exposure is a potential cause of increased risk of hospitalization for urolithiasis. In recent years, air pollution is an important factor causing kidney disease, mainly because the kidney is susceptible to the toxic effects of air pollutants (Xu et al. 2018). One study (Chen et al. 2018) found that NO_2_ was associated with a lower glomerular filtration rate and a higher prevalence of chronic kidney disease. Existing studies have also confirmed that NO_2_ is a major atmospheric pollutant affecting the hospitalization rate of chronic kidney disease (Wu et al. 2020; Ye et al. 2021). In addition, NO_2_ is correlated to the occurrence of oxidative stress. More and more studies have confirmed that oxidative stress is strongly correlated with the formation of kidney stones (Jeong et al. 2020; Wang et al. 2022), mainly because it can maintain an inflammatory microenvironment, reshape the extracellular matrix, and lead to the accumulation of crystal deposits, thus promoting kidney stone formation (Wigner et al. 2021). In conclusion, although the exact biological pathways by which air pollution induces kidney injury are not fully understood, it is possible that air pollution-related renal toxicity is caused by the same biological mechanisms through which air pollution exerts a negative impact on the cardiovascular system, including oxidative stress and systemic inflammation, blood pressure changes, or vascular damage (Rasking et al. 2022). Therefore, the correlation between NO_2_ and urolithiasis needs to be further explored.

Our dual-pollutant model demonstrated that after adjusting for PM_2.5_, PM_10_, SO_2_, CO, and O_3_, the association between NO_2_ (day 1 lag) and number of hospitalizations for urolithiasis remained robust. In addition, PM_2.5_, PM_10_, SO_2_, CO, and O_3_ could increase the risk of hospitalization of urolithiasis when the cumulative lag was 3 days. This further suggests a strong association between NO_2_ exposure and hospitalizations for urolithiasis. It has been reported that ambient PM and NO_2_ may contribute to kidney damage through peroxidation. It has also been shown that factors affecting the formation of urolithiasis include PM_2.5_, potentially by affecting serum creatinine and uric acid levels (Aizezi et al. 2022). In addition, studies have shown that O_3_ exposure is related to elevated levels of systemic inflammation (Arjomandi et al. 2015), related to the up-regulation of redox homeostasis in vivo caused by long-term continuous exposure to O_3_ (Hu et al. 2020). Moreover, it has been shown that high concentration of environmental PM and O_3_ exposure could lead to inflammation and oxidative stress in the perirenal adipose tissue of rats (Sun et al. 2013). Finally, an experimental study in mice exposed to PM_2.5_ for a long time revealed increased levels of DNA damage related to renal oxidative stress (Bowe et al. 2020). Therefore, based on previous studies, we speculate that the effect of air pollutants on urolithiasis may be related to increased levels of oxidative stress and inflammation in the body. Future studies should further explore this pathogenic mechanism.

According to the attribution score and quantitative analysis, the average length and the average cost of hospitalization were 16 days and 21,164.39 RMB, respectively. It is estimated that up to 5.75% of the total hospitalizations for urolithiasis can be attributed to NO_2_, and the cost of hospitalizations for urolithiasis related to NO_2_ is about 3,430,000 RMB. In addition, there is a large difference in the incidence rate of urolithiasis between male and female, with women having a significantly higher incidence rate compared to men. However, it is worth noting that even though the prevalence rate of female is higher than that of male, the number of hospitalizations, length of stay, and hospitalization cost of men are higher than those of women. This result indicates that men have a certain economic burden and human and material loss due to urolithiasis. Similarly, in terms of age groups, we concluded that the prevalence of urolithiasis was higher in those aged ≥65 years compared to people aged <65 years, but the number of hospital days, hospital admissions, and hospital costs after urolithiasis were greater in those aged <65 years compared to those aged ≥65 years. These findings suggest that people younger than 65 spend more time, energy, and money on urolithiasis. In addition, different populations are more susceptible to urolithiasis during warm seasons than during cold seasons. Therefore, based on in this study, the relevant government departments should strengthen health education on urolithiasis to improve the public’s awareness of the disease. At the same time, the relevant environmental departments should also strengthen public awareness regarding environmental protection, in order to reduce the morbidity and economic losses caused by environmental pollution.

We conducted a stratified analysis based on age, sex, and season. The results showed that with increasing NO_2_ concentrations, age 65 years or older was more strongly correlated with hospitalizations for urolithiasis than age younger than 65 years. In terms of age effects, elderly individuals (older than 65 years of age) are more susceptible to air pollutants than other age groups because of their weaker immune systems (Tong et al. 2016). The results of this study showed that the risk of hospitalization for urolithiasis increases with increasing NO_2_ concentrations, and this effect was stronger for females than for males. This finding is consistent with that of another study (Safdar et al. 2021). Other studies have also shown that females and individuals older than 65 years of age seem to be more susceptible to the effects of air pollution than males and younger individuals (Manisalidis et al. 2020). Previous studies have suggested that males are 2 to 3 times more likely to develop stones than females; however, data from recent studies suggest that this difference is decreasing (Khan et al. 2016). The adverse effects of air pollution and their association with sex differences have rarely been the focus of scholarly research. Sex differences in terms of behavior, occupation, and social/family roles cause differences in air pollution exposure, which may explain the variability in the risk associated with air pollution exposure of males and females (Matz et al. 2014). However, the current limited understanding of biological mechanisms presents a challenge when explaining the association between sex differences and air pollution in terms of its effect on the risk of hospitalization for urolithiasis. Additionally, our study found a stronger correlation between warm seasons and hospitalizations for urolithiasis with increasing NO_2_ concentrations compared to cold seasons. Human sensitivity to air pollutants may vary with seasons because of physiological differences in hormones, biology, and structure/morphology. Another study (Shin et al. 2022) found seasonal differences in hospitalizations for circulatory and respiratory diseases and mortality rates in response to short-term exposure to air pollutants. However, reports related to urolithiasis are scarce. Therefore, more data from different regions are necessary to further explore the correlation between age, sex, and season, and the number of hospitalizations for urolithiasis as air pollutant concentrations increase.

The study was stratified by age, sex, and season, which could help target specific interventions to sensitive groups. However, several limitations of our study should be noted. First, air pollution data were obtained from fixed site monitors. Although errors are inevitable, the resulting nondifferential errors may have resulted in the underestimation of the impact of air pollutants (Richmond and Long 2020). Second, this study focused on the average daily temperature. Further studies should consider different temperature indicators, such as extreme and daily maximum temperatures. Finally, the relatively low daily number of urolithiasis cases may have caused us to miss important associations between air pollutants and urolithiasis, especially in subgroup analyses. Future multicenter studies with longer durations are required to validate these results.

## Conclusions

Taken together, the results of this study suggest that short-term exposure to NO_2_ is associated with an increased risk of hospitalization for urolithiasis. Among these hospitalizations, women and the elderly are more susceptible to air pollution, especially NO_2_. Furthermore, the number of hospitalizations for urolithiasis is higher during the warm season. Finally, men and people younger than 65 years of age incur more costs for urolithiasis-related hospitalizations. Further studies are needed to confirm our findings and the underlying mechanisms involved.

## Supplementary information

ESM 1

## Data Availability

The datasets used and/or analyzed during the current study are available from the corresponding author on reasonable request.
