# Supplementary material for: Association of short-term nitrogen dioxide exposure with hospitalization for urolithiasis in Xinxiang, China: a time series study
Source: Environ Sci Pollut Res Int. 2023 Jul 29;30(41):93697–707. doi: 10.1007/s11356-023-28539-0 (PMC10468926; doi:10.1007/s11356-023-28539-0)
Supplement: Supplementary file 1 — ESM 1 [file 11356_2023_28539_MOESM1_ESM.docx]

**Supplemental Materials**

**Manuscript title:** Association of Short-term Nitrogen Dioxide Exposure with Hospitalization for Urolithiasis in Xinxiang, China: A Time Series Study
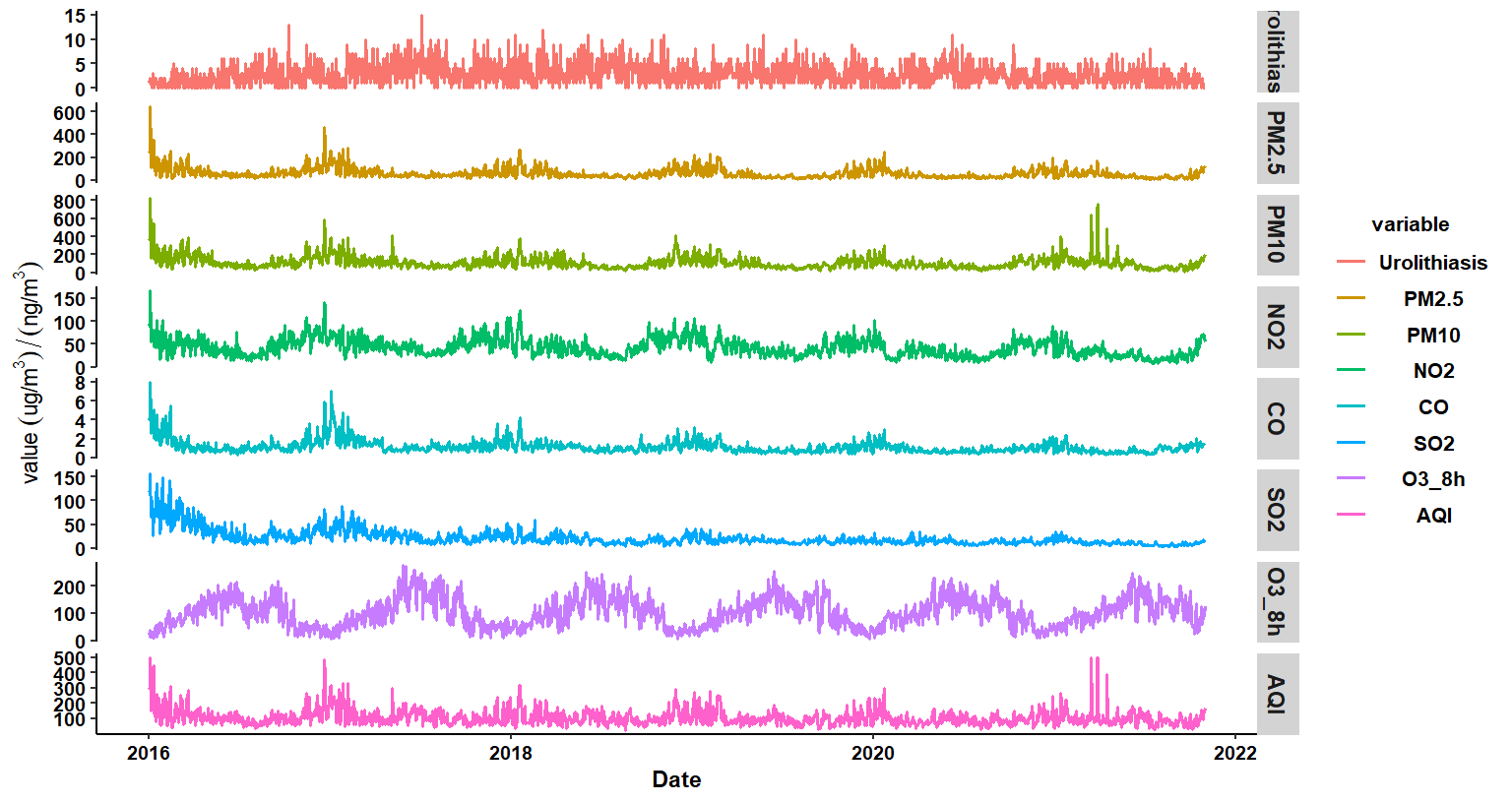


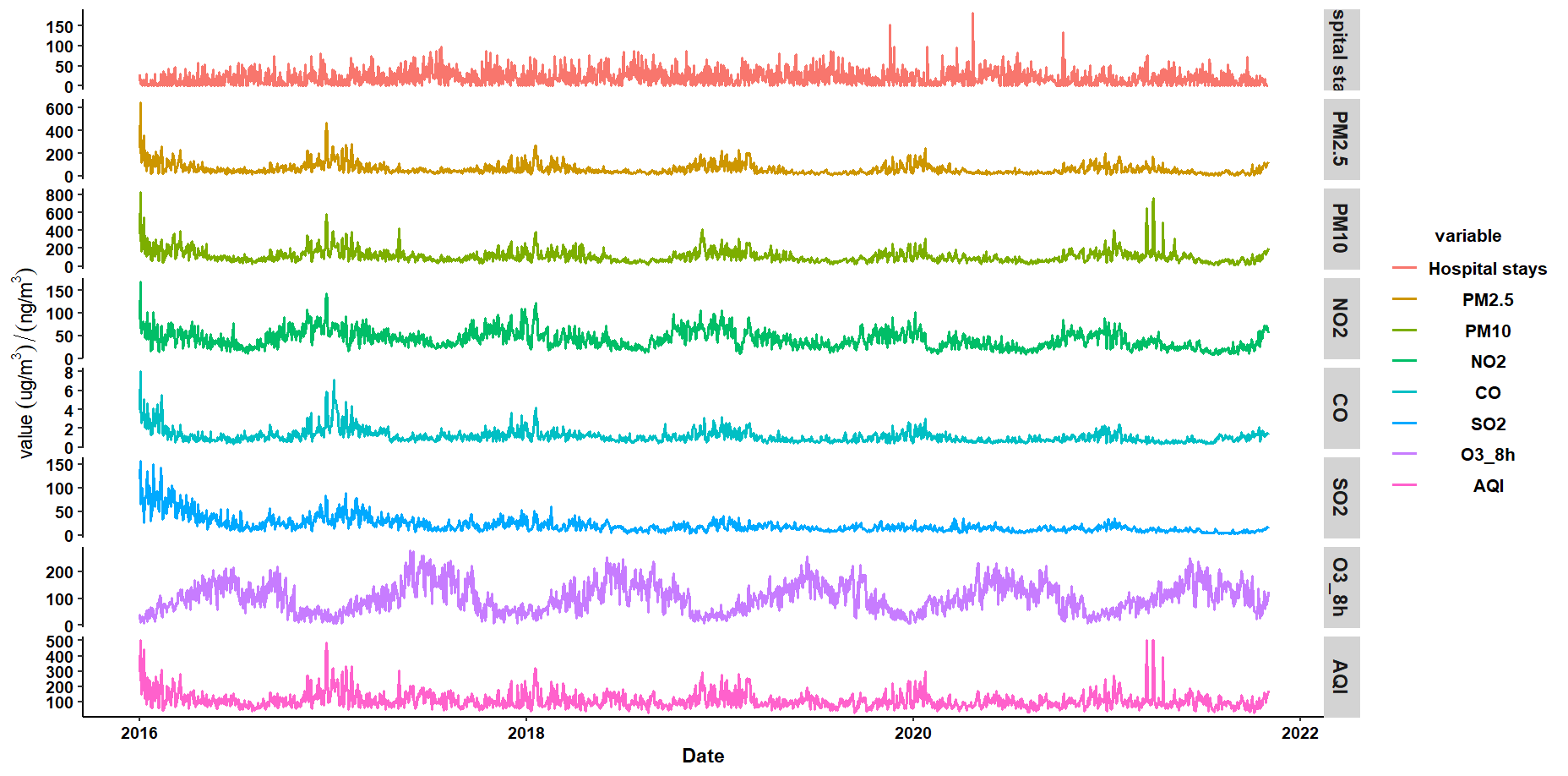


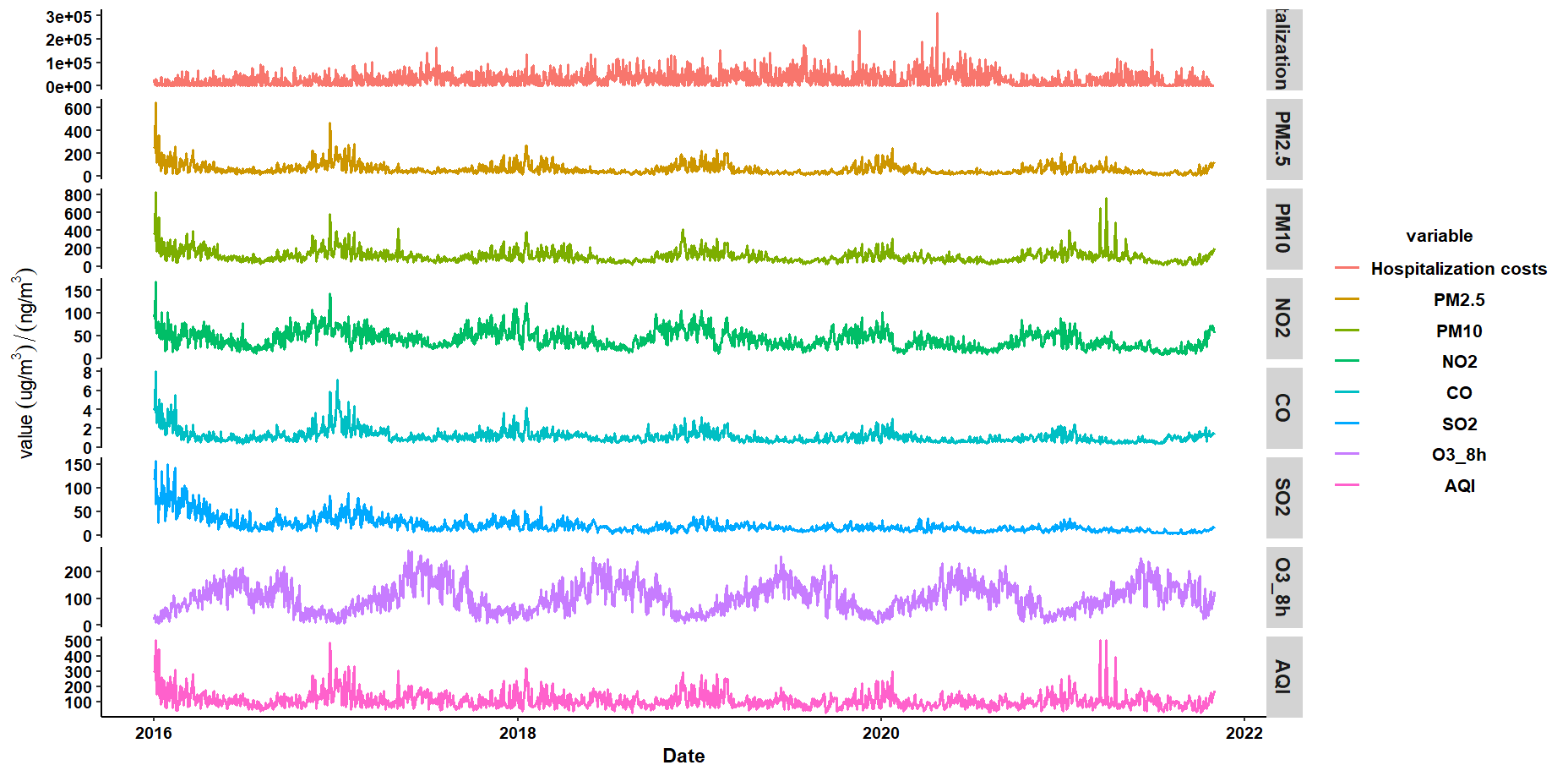


Fig S1. The distribution of meteorological variables, ambient air pollutant concentrations, and daily urolithiasis hospitalizations, hospital stays and hospitalization costs in Xinxiang, China from 2016 to 2021.


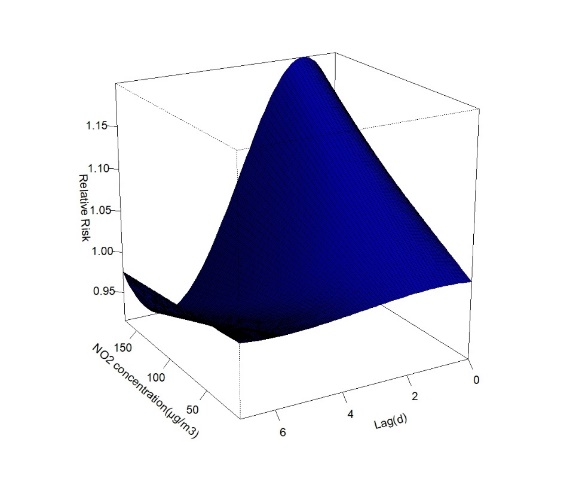


Fig S2. Three-dimensional correlation plot of the effect of NO_2_ on urolithiasis hospitalizations at different lag times.


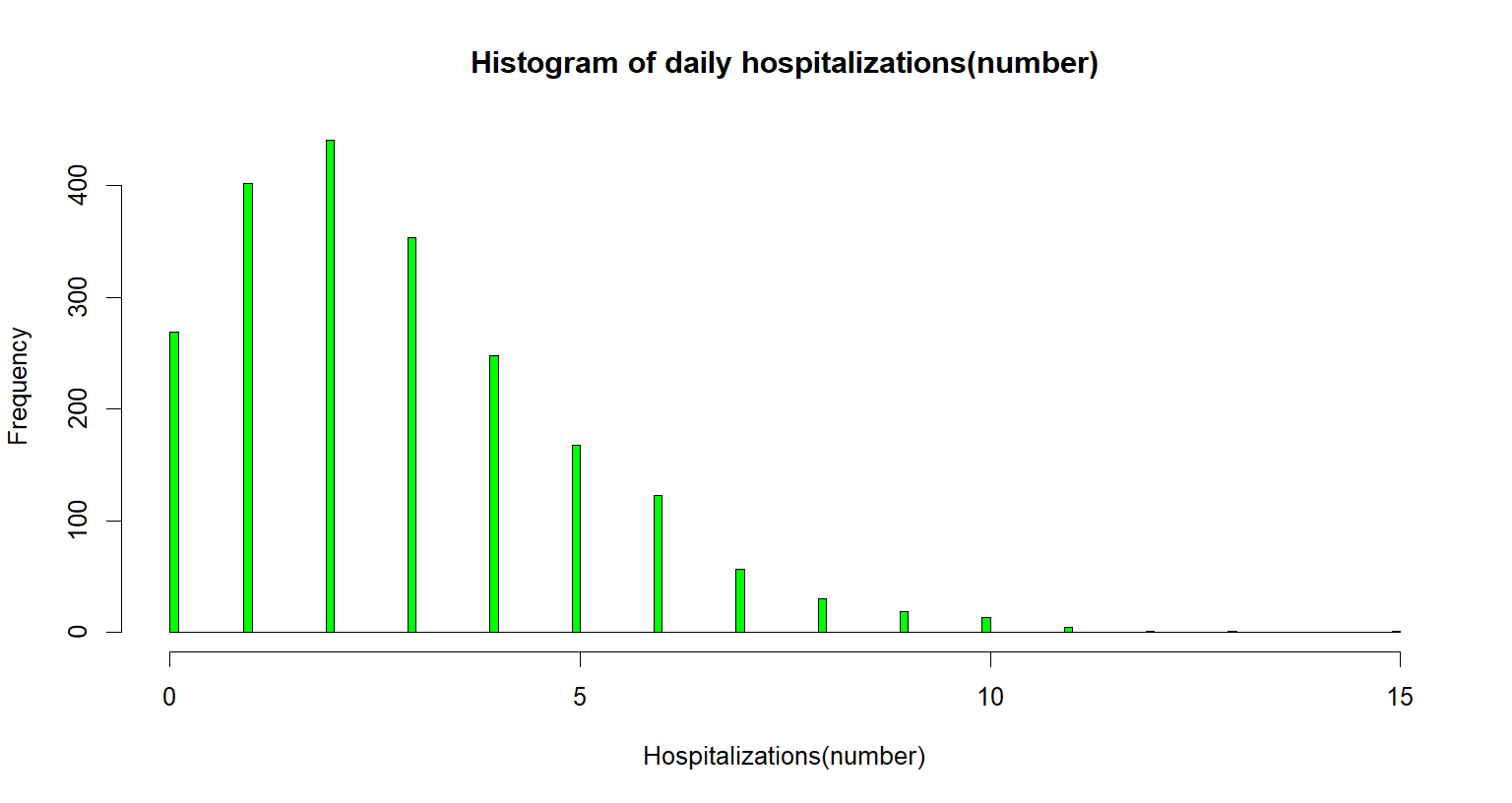


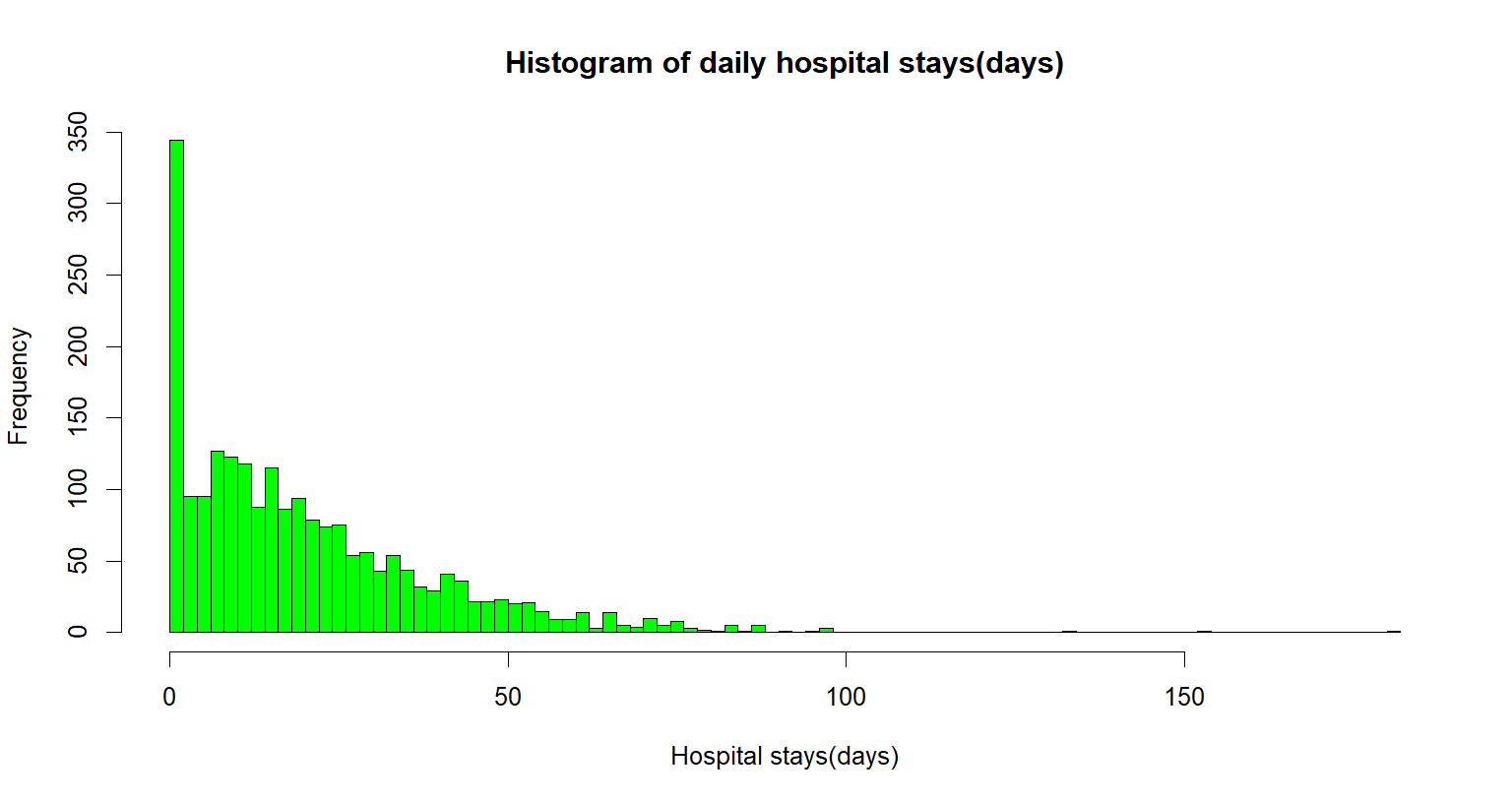


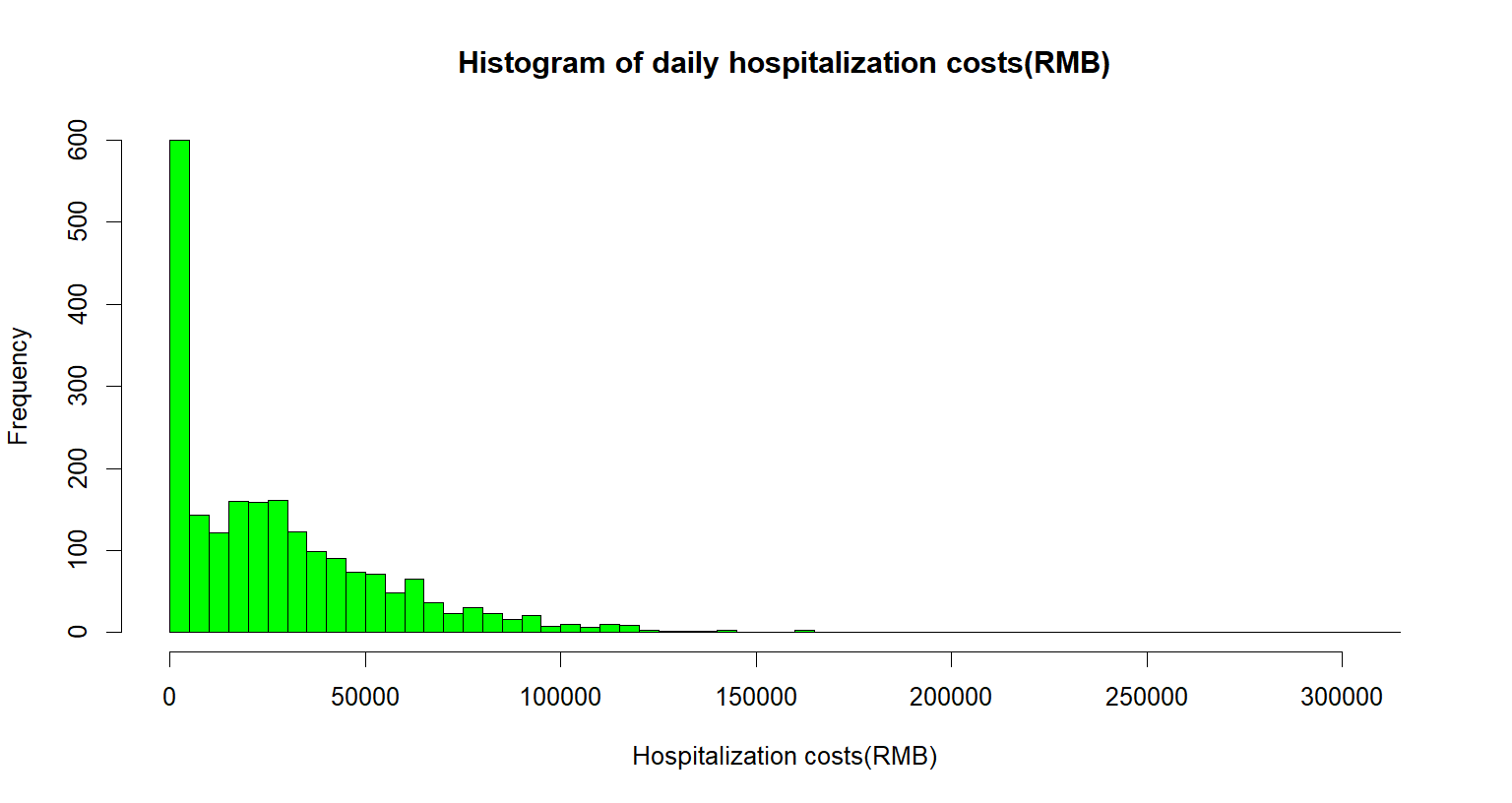


Fig S3. The distribution of daily urolithiasis hospitalizations, hospital stays, and hospitalizations costs.

| Table S1. 95% CIs of Urolithiasis hospitalizations associated with a 10 μg/m^3^ increment in NO_2_ concentration at different lag days in age-stratified analyses. | | |
| --- | --- | --- |
| LAG days | Age＜65 years old | Age≥65 years old |
| lag0 | 1.016 (0.975-1.057) | 1.021 (0.917-1.138) |
| lag1 | 1.014 (0.995-1.035) | **1.074 (1.019-1.131)** |
| lag2 | 1.011 (0.988-1.034) | **1.074 (1.012-1.139)** |
| lag3 | 1.006 (0.988-1.024) | 1.043 (0.996-1.093) |
| lag4 | 1.001 (0.983-1.019) | 1.004 (0.958-1.052) |
| lag5 | 0.996 (0.974-1.019) | 0.977 (0.920-1.037) |
| lag6 | 0.994 (0.975-1.014) | 0.980 (0.931-1.030) |
| lag7 | 0.995 (0.958-1.034) | 1.033 (0.935-1.142) |
| lag01 | 1.030 (0.981-1.082) | 1.097 (0.962-1.249) |
| lag02 | 1.042 (0.988-1.098) | **1.178 (1.024-1.355)** |
| lag03 | 1.048 (0.990-1.109) | **1.229 (1.057-1.428)** |
| lag04 | 1.048 (0.988-1.113) | **1.234 (1.054-1.445)** |
| lag05 | 1.045 (0.979-1.115) | **1.205 (1.014-1.433)** |
| lag06 | 1.039 (0.967-1.115) | 1.181 (0.976-1.428) |
| lag07 | 1.034 (0.960-1.113) | **1.220 (1.000-1.488)** |

| Table S2. 95% CIs of Urolithiasis hospitalizations associated with a 10 μg/m^3^ increment in NO_2_ concentration at different lag days in sex-stratified analyses. | | |
| --- | --- | --- |
| LAG days | Male | Female |
| lag0 | 0.999 (0.954-1.046) | 1.043 (0.980-1.111) |
| lag1 | 1.013 (0.990-1.036) | **1.033 (1.001-1.065)** |
| lag2 | 1.016 (0.991-1.042) | 1.018 (0.983-1.055) |
| lag3 | 1.012 (0.992-1.033) | 1.003 (0.976-1.031) |
| lag4 | 1.004 (0.984-1.025) | 0.991 (0.964-1.019) |
| lag5 | 0.996 (0.971-1.022) | 0.986 (0.952-1.022) |
| lag6 | 0.991 (0.969-1.012) | 0.991 (0.961-1.021) |
| lag7 | 0.991 (0.949-1.035) | 1.009 (0.951-1.071) |
| lag01 | 1.012 (0.958-1.070) | 1.077 (0.999-1.161) |
| lag02 | 1.028 (0.969-1.092) | **1.097 (1.011-1.189)** |
| lag03 | 1.041 (0.976-1.110) | **1.100 (1.008-1.201)** |
| lag04 | 1.046 (0.977-1.119) | 1.090 (0.994-1.196) |
| lag05 | 1.041 (0.967-1.121) | 1.075 (0.972-1.190) |
| lag06 | 1.032 (0.951-1.118) | 1.065 (0.953-1.191) |
| lag07 | 1.022 (0.940-1.112) | 1.075 (0.957-1.207) |

| Table S3. 95% CIs of Urolithiasis hospitalizations associated with a 10 μg/m^3^ increment in NO_2_ concentration at different lag days in season-stratified analyses. | | |
| --- | --- | --- |
| LAG days | Warm season | Clod season |
| lag0 | 0.997 (0.925-1.077) | 1.012 (0.950-1.078) |
| lag1 | **1.052 (1.009-1.097)** | 1.000 (0.971-1.031) |
| lag2 | **1.065 (1.020-1.113)** | 0.994 (0.962-1.027) |
| lag3 | **1.051 (1.013-1.089)** | 0.991 (0.966-1.017) |
| lag4 | 1.024 (0.987-1.061) | 0.990 (0.965-1.015) |
| lag5 | 0.999 (0.957-1.043) | 0.988 (0.957-1.021) |
| lag6 | 0.990 (0.952-1.030) | 0.985 (0.958-1.012) |
| lag7 | 1.011 (0.941-1.087) | 0.977 (0.925-1.032) |
| lag01 | 1.050 (0.949-1.160) | 1.012 (0.937-1.093) |
| lag02 | 1.118 (0.995-1.256) | 1.006 (0.926-1.092) |
| lag03 | **1.175 (1.029-1.341)** | 0.997 (0.914-1.088) |
| lag04 | **1.202 (1.039-1.391)** | 0.987 (0.901-1.081) |
| lag05 | **1.201 (1.021-1.412)** | 0.975 (0.883-1.077) |
| lag06 | 1.189 (0.995-1.422) | 0.960 (0.859-1.072) |
| lag07 | 1.203 (0.991-1.459) | 0.938 (0.833-1.057) |
